# A Comprehensive Review of the Application of Bacteriophages Against Enteric Bacterial Infection in Poultry: Current Status, Challenges, and Future Prospects

**DOI:** 10.3390/antibiotics14121207

**Published:** 2025-12-01

**Authors:** Muhammad Muneeb, Ehsaan Ullah Khan, Sohail Ahmad, Ijaz Hussain, Shumaila Batool, Arooj Fatima, Elham Assadi Soumeh, Ali R. Al Sulaiman, Ala E. Abudabos

**Affiliations:** 1 Department of Animal Nutrition, Faculty of Animal Production and Technology, University of Veterinary and Animal Sciences, Lahore 54000, Pakistan; 2022-mphil-1331@uvas.edu.pk (M.M.); ehsaan@uvas.edu.pk (E.U.K.); 2022-mphil-1332@uvas.edu.pk (I.H.); 2 Department of Poultry Production, Faculty of Animal Production and Technology, University of Veterinary and Animal Sciences, Lahore 54000, Pakistan; 3 Department of Zoology, Wildlife and Fisheries, University of Agriculture, Faisalabad 38000, Pakistan; 2022ag1625@uaf.edu.pk; 4 Department of Veterinary Medicine, Faculty of Veterinary Sciences, University of Veterinary and Animal Sciences, Lahore 54000, Pakistan; 2022-mphil-1420@uvas.edu.pk; 5 School of Agriculture and Food Sustainability, The University of Queensland, Gatton, QLD 4343, Australia; e.soumeh@uq.edu.au; 6 Environmental Protection Technologies Institute, Sustainability and Environment Sector, King Abdulaziz City for Science and Technology, P.O. Box 6086, Riyadh 11442, Saudi Arabia; arsuliman@kacst.gov.sa; 7 Department of Food and Animal Sciences, College of Agriculture, Tennessee State University, Nashville, TN 37209, USA

**Keywords:** bacteriophage, enteric disease, poultry, antibiotic alternative, phage therapy

## Abstract

The poultry industry plays a major role in the emergence and spread of foodborne zoonotic diseases, particularly those associated with antibiotic-resistant bacteria. These diseases pose substantial global public health challenges, and the increasing development of antimicrobial resistance further intensifies these concerns. In response, scientific efforts have expanded to develop and implement innovative technologies capable of mitigating the rising prevalence of multidrug-resistant (MDR) microorganisms. Therapeutic bacteriophage supplementation has regained significant attention because it can selectively lyse specific bacteria, is cost-effective to produce, offers environmentally favorable characteristics, and provides several advantages over conventional antibiotics. Experimental studies have demonstrated that phage therapy is both safe and effective for controlling poultry-associated enteric pathogens. Phages can be applied at various stages of the poultry production chain, from rearing to processing and distribution, using multiple delivery strategies. Despite certain limitations, the targeted and well-regulated application of phage cocktails offers considerable potential as an alternative to antibiotics for managing MDR infections. The success of bacteriophage therapy depends on several factors, including the timing of administration, dosage, delivery method, and its integration with other therapeutic approaches. Therefore, developing a comprehensive understanding of bacteriophage utilization in poultry production is both timely and necessary. This review examines the applications, constraints, and future opportunities of phage therapy within the commercial poultry industry, with particular emphasis on the mechanisms through which bacteriophages control bacterial infections.

## 1. Introduction

The poultry industry is recognized as one of the fastest-growing sectors in the global food market [1]. In recent years, the sector has expanded significantly, driven primarily by cost-efficient production systems and rising consumer demand for poultry meat [2]. Advancements in genetic selection for rapid growth, housing systems, and feed formulation have contributed to higher meat yield, improved feed conversion ratios (FCR), and reduced mortality rates [3]. A bird’s nutritional status and overall health are closely linked to its gut condition. Chicken gut health encompasses the gut immune system, the balance of the gut microbiota, and the structural integrity of the gastrointestinal tract (GIT) at both macro- and microscopic levels. GIT health plays a critical role in several physiological functions, including nutrient digestion, absorption, metabolism, disease resistance, and gut-associated immune responses [4]. Disruptions to these processes can lead to intestinal disorders, resulting in substantial declines in flock performance.

Enteric diseases present a major challenge to the poultry industry. They cause substantial economic losses through reduced weight gain, poor FCR, increased mortality, higher treatment costs, and a heightened risk of product contamination. As a result, these conditions undermine the sustainable consumption of poultry products [5]. A wide range of pathogenic agents, including bacteria, parasites, viruses, and other infectious or non-infectious factors, have been identified as causes of enteric disorders, either independently or in combination [6]. Gastrointestinal disorders encompass several conditions, such as dysbacteriosis, malabsorption syndrome, wet droppings, salmonellosis, campylobacteriosis, the intestinal form of colibacillosis, coccidiosis, and necrotic enteritis [7]. The poultry industry spends an estimated £7.7–13.0 billion annually across seven countries on prevention, treatment, and productivity losses related to avian coccidiosis [8]. Necrotic enteritis also imposes a severe economic burden, with global losses estimated at USD 6 billion per year [9]. Colibacillosis is a major cause of mortality, reaching up to 20%, and morbidity in poultry. It reduces meat yield (with a 2% decrease in live weight and a 2.7% decline in FCR), lowers egg production by up to 20%, decreases hatchability, and increases carcass condemnation rates to as high as 43% at slaughter [10]. All age groups of chickens are susceptible and reported prevalence ranges from 9.52% to 36.73% [11]. In addition, *Salmonella*-contaminated animal products account for roughly 3% of global bacterial foodborne diseases, causing an estimated 80 million illnesses and 155,000 deaths worldwide [12].

Antibiotics used as antimicrobial growth promoters (AGPs) have helped control enteric diseases to some extent [13]. However, the continual and unregulated use of antibiotics for both growth promotion and therapeutic purposes has allowed enteric pathogens to develop resistance to many commonly used antimicrobial agents [14]. As antibiotic resistance continues to emerge, the therapeutic application of antibiotics has attracted increasing attention from consumers, regulatory bodies, and researchers [15]. AGPs in poultry production have been gradually banned or restricted in many countries, including the European Union, Canada, the United States, Hong Kong, and Japan [16,17]. Countries that have limited the use of AGPs have subsequently experienced a marked rise in economically significant enteric diseases [18,19,20]. In parallel, there has been growing interest in feasible alternative strategies to maintain animal health and ensure efficient livestock production [17,21]. Proposed alternatives include probiotics, prebiotics, synbiotics, postbiotics, phytobiotics, enzymes, organic acids, nanoparticles, and bacteriophages [22]. These AGPs substitute function by modulating the intestinal microbiota, stimulating immune responses, and suppressing pathogenic organisms. Furthermore, improved management practices and vaccines have also become the focus of research as potential disease-control strategies [23,24]. Identifying a single alternative that is effective under all production conditions remains difficult. While some strategies substantially reduce the prevalence of enteric infections, they may also increase production costs. Therefore, effective control of enteric diseases generally requires the simultaneous use of multiple complementary approaches.

Bacteriophages (phages) are viruses that specifically target and infect prokaryotic organisms, including bacteria and archaea [25]. They possess a protein structure called a capsid, which consists of a head and a tail, and encloses a nucleic acid molecule, either DNA or RNA. The tail is a complex structure that plays a critical role in bacterial recognition, attachment, cell entry, and genome delivery. The capsid protects the phage’s genetic material throughout this process. Bacteriophages first attach to the surface of a bacterial cell and then inject their genetic material into the host. Once inside, they replicate within the host cytoplasm until the infected cell lyses. The newly released virions can subsequently infect neighboring bacteria, thereby directly influencing bacterial populations [26,27].

Phages can also influence the host’s immune system. They affect innate immunity by modulating phagocytosis and cytokine responses, and they impact adaptive immunity by altering antibody production [28]. Phages are classified as either lytic, undergoing the lytic cycle, or temperate, undergoing the lysogenic cycle, based on their interaction with bacteria and the progression of their life cycle [29]. Bacteriophages exhibit high specificity, targeting either a single bacterial species (monovalent) or closely related species (polyvalent) [22]. This specificity is determined by factors such as the presence of cell surface receptors, the outer membrane, lipopolysaccharides, and flagella [30]. Because of this high degree of specificity, phage therapy offers a targeted approach that eliminates harmful bacteria while preserving beneficial members of microbiota. As a result, it reduces the risk of secondary infections commonly associated with broad-spectrum antibiotics and minimizes disruption to the host’s microbial balance. Moreover, phages have demonstrated effectiveness against both antibiotic-susceptible and antibiotic-resistant bacterial strains, making them a promising alternative or adjunct to conventional antibiotic treatments [31].

**Figure 1:**
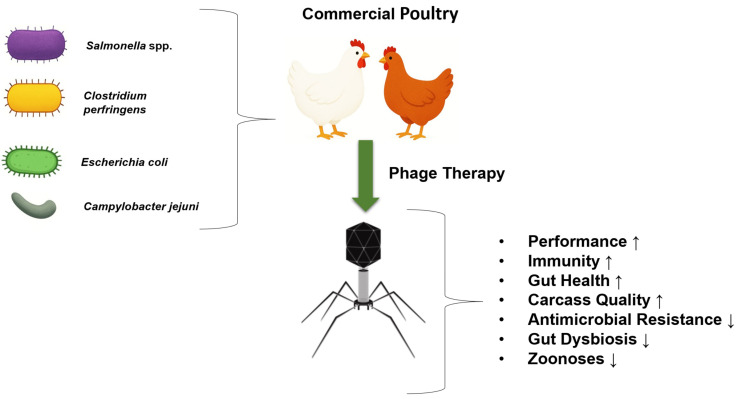
Applications of bacteriophages against enteric bacterial diseases in poultry production.

The efficacy of bacteriophages in controlling bacterial infections and reducing bacterial populations in chickens has been extensively investigated [22]. This review provides a comprehensive overview of the effects of various phages on gut-health parameters, microbial load, production performance, blood components, immune responses, and post-harvest meat safety in the poultry industry, particularly in relation to enteric diseases of major public-health concern (Figure 1). The information presented here offers a useful resource for understanding the potential of various bacteriophages as alternative therapeutic agents to mitigate the adverse impacts of enteric disorders in poultry.

## 2. Background of Bacteriophages

Bacteriophages were independently discovered by Frederick Twort and Félix d’Herelle [32,33,34]. Twort reported a “glassy transformation” in micrococcal colonies, whereas d’Herelle isolated an “antimicrobe” from *Shigella* and introduced the term *bacteriophage*, meaning “bacteria eater.” Bacteriophages represent the most abundant biological entities in the biosphere, with an estimated population of 4.8 × 10^31^ particles (approximately one order of magnitude lower than the total number of bacterial cells on Earth) [35]. These viruses are ubiquitous across all environments inhabited by bacteria, including aquatic, terrestrial, botanical, and various food-related ecosystems. Consequently, human exposure to bacteriophages is common and generally considered harmless, as they are not viewed as a threat to human health.

Phages are increasingly recognized as important components of the human innate microbiota. In the human gut virome, bacteriophages, collectively referred to as the phageome, constitute the dominant group. However, the dynamics of the intestinal phageome in chickens with a balanced gut microbiota remain largely unknown [36].

## 3. Life Cycle of Bacteriophages

Bacteriophage biology has been extensively studied for more than a century. Bacteriophages fall into two main categories, lytic and temperate (lysogenic) [37], which differ in how they interact with their bacterial hosts [38]. Attachment to the bacterial cell via specific proteins is essential for the initiation of the bacteriophage life cycle.

### 3.1. Lytic Cycle

Bacteriophages replicate within host cells through a six-stage process known as the lytic cycle. The cycle begins when the phage attaches to specific surface proteins on the bacterial cell using its tail fibers. The tail then contracts and injects the phage DNA or RNA into the host, leaving the empty capsid outside. Once inside, the phage genome commandeers the host’s cellular machinery and directs both host and viral enzymes to synthesize viral proteins and replicate the phage genome. Each new virion is assembled from three major protein components that form the head, tail, and tail fibers. During head formation, the replicated genome is packaged into the capsid. In the final stage, the phage expresses genes encoding endolysin, an enzyme that degrades the host cell wall and allows external fluid to enter. This influx causes the cell to swell excessively. Eventually, the cell lyses, releasing newly formed phage particles that can infect neighboring bacterial cells [29,39]. The entire lytic cycle may last from 2 min to 2 h.

### 3.2. Lysogenic Cycle

The lysogenic cycle differs from other viral replication processes because it does not necessarily cause immediate lysis of the bacterial cell. The bacteriophage first attaches to the host cell surface and injects its DNA or RNA. Rather than directing the synthesis of new viral particles, the phage genome integrates into the bacterial chromosome, forming a prophage. This prophage is replicated passively as the bacterium reproduces and is transmitted to its daughter cells. Over time, this process generates a large population of bacteria carrying the prophage [40]. Under stressful or otherwise inducing conditions, the prophage may excise itself from the bacterial chromosome and initiate the lytic cycle [41].

## 4. Applications of Bacteriophages in the Poultry Industry

Bacteriophages are also being investigated and applied within the poultry industry. They can be used in multiple aspects of poultry farming, production, and processing [22]. The applications of bacteriophages across different segments of the poultry sector are outlined below.

### 4.1. Use of Phages as Feed Additives in Commercial Poultry

Phage therapy has proven to be an effective feed supplement for enhancing animal performance and health. Studies across various livestock species indicate that phage supplementation can reduce the colonization of pathogenic bacteria in the GIT, thereby improving growth rates and lowering the risk of food spoilage [42,43]. In addition, phages can modulate gut microbiota, support gastrointestinal health, enhance nutrient absorption in intensive production systems, and serve as viable alternatives to in-feed antibiotics [44,45,46].

The mode of delivery plays a critical role in the effectiveness of phages in livestock, with the best outcomes achieved through direct administration at the site of infection. A systematic review and meta-analysis by Mosimann et al. [47] found that delivering phages to chickens through feed was more effective than administering them via drinking water or aerosol spray. Similarly, Oliveira et al. [48] reported that lytic coliphages can be administered orally or by spray to treat respiratory *Escherichia coli* (*E. coli*) infections in broiler chickens.

Phage deactivation by environmental factors, effective delivery, dosage maintenance, and sustained release at the infection site all rely on proper formulation, stability, and encapsulation methods such as emulsification, polymerization, extrusion dripping, and spray-drying [49,50]. Ma et al. evaluated both free and orally encapsulated phages to examine their viable distribution and prolonged release within the GIT of chickens. The results showed that free phages moved through the tract more rapidly, as indicated by the increased fecal concentration observed after 1.5 h. In contrast, orally encapsulated phages required more than four hours to pass completely through the GIT of young chickens [51].

Phages are highly sensitive to low pH of stomach (proventriculus and gizzard) and intestinal enzyme activity, which limits their effectiveness in treating bacterial infections and improving animal performance. In this context, some previous studies evaluated the efficacy of an oral phage treatment in broiler chickens, comparing three phages encapsulated in alginate/CaCO_3_ with the same unencapsulated phages against *Salmonella*. The findings showed that the encapsulated phages demonstrated enhanced and prolonged activity, indicating that encapsulation protects them from gastric acidity in the GIT [52].

### 4.2. Bacteriophages—Emerging Antibacterial Agents in Poultry Farming

Public health concerns have intensified attention on enteric poultry pathogens, particularly *Campylobacter jejuni* (*C. jejuni*), *Salmonella enterica* serovar Enteritidis (*S. Enteritidis*), *Salmonella enterica* serovar Typhimurium (*S. Typhimurium*), *E. coli*, and *Clostridium perfringens* (*C. perfringens*), because of their potential transmission through poultry-derived food products.

After *Campylobacter*, the second most significant zoonotic foodborne pathogen is *Salmonella*, which has a substantial impact on commercial poultry production. In the EU, *S. Enteritidis* accounts for most reported human salmonellosis cases. Poultry meat also shows the highest proportion of *Salmonella*-positive samples. Although the prevalence of *Salmonella* serovars affecting broilers, laying hens, breeding hens, and fattening turkeys has declined in recent years within the EU, it has remained stable in breeding turkeys [53]. Currently, numerous bacteriophage-based products are commercially available to prevent *Salmonella* infections in various animal species, including poultry [54,55].

*Escherichia coli* is a Gram-negative bacillus commonly present in the GIT of birds and transmitted through fecal contamination. Most strains are harmless; however, certain pathogenic serotypes, collectively known as avian pathogenic *E. coli* (APEC), can cause disease, leading to mortality and carcass condemnations. This opportunistic pathogen is capable of causing both primary and secondary infections and is frequently associated with disease in poultry across all ages and production types. Food-borne pathogenic groups such as enterohemorrhagic *E. coli* (EHEC) and its subgroup, Shiga toxin–producing *E. coli* (STEC), are responsible for severe human illnesses worldwide [56]. Bacteriophages that infect *E. coli* are termed coliphages. Although only a few phage-based products targeting colibacillosis in poultry are currently available commercially, active research in this area is ongoing.

*Campylobacter* species are widespread in nature and commonly inhabit the GIT of birds as commensals. Poultry, in particular, serves as a natural reservoir for Campylobacter, largely because its elevated body temperature promotes bacterial colonization [57]. These bacteria are major contributors to foodborne illness in humans and are frequently detected in slaughterhouses and poultry products worldwide [51]. Phage therapy has demonstrated strong effectiveness in reducing *Campylobacter* colonization in broilers, thereby lowering the risk of transmission through the food chain. However, no commercial phage preparation is currently available to control *Campylobacter* infections, despite an urgent need for alternative interventions. *Campylobacter* phages possess distinctive biological characteristics that make them more difficult to apply than other lytic phages. Key challenges include difficulties in phage isolation, propagation, and purification. Although *Campylobacter* phages are genetically similar, considerable heterogeneity exists among populations in terms of lytic activity, host range, and infection kinetics, complicating the identification of suitable candidates. An additional concern is the emergence of phage-resistant *Campylobacter* strains, reported at rates of 1–14%. Finally, the high cost of large-scale phage production continues to impede commercial development [27,58].

*Clostridium perfringens* is a Gram-positive, rod-shaped, non-motile, spore-forming, anaerobic bacterium. It is widespread in the natural environment and is a common component of the poultry gut microbiota. Although generally non-pathogenic at low levels (<10^4^ CFU), its pathogenicity is primarily associated with toxin production. Toxins produced by *C. perfringens* types A, C, and G can cause both acute and subclinical forms of necrotic enteritis, one of the most economically significant diseases in poultry. In humans, foodborne illness may result from the consumption of poultry meat contaminated with enterotoxin-positive *C. perfringens* strains [15]. Several *C. perfringens* strains have shown resistance to bacteriophages [59,60]. Many researchers propose that phage-encoded endolysins may be particularly effective for controlling this species. Findings indicate that endolysins can act against all tested *C. perfringens* strains, although variations in strain susceptibility have been observed [51,61,62].

Table 1, Table 2, Table 3 and Table 4 summarize studies examining the use of bacteriophages as alternatives to antibiotics in chickens. These tables concisely present the phage cocktail types, administration methods, dosage levels, target infections, and chicken breeds involved. They also outline the effects of each phage preparation on performance, intestinal health, and blood parameters. The effectiveness of phage therapy in improving animal health and performance depends on multiple variables, including delivery method, concentration, phage efficacy, treatment duration, bird age, treatment type, experimental design, timing of administration, preparation protocol, and the bacterial species targeted [47]. Additional considerations include sample collection procedures, phage resistance, phage isolation techniques, pH, phage neutralization, temperature, accessibility to the target bacteria, and the phage-to-bacteria ratio [63].

#### 4.2.1. Influence of Bacteriophage Use on Microbial Load

Bacteriophage treatments have been shown to enhance gut health in poultry by reducing pathological lesions and improving intestinal histology. Phage therapy is an effective strategy against infections because it selectively targets pathogenic bacteria. Table 1 summarizes the efficacy of bacteriophage applications in reducing pathogenic microbial loads in the GIT and internal organs of poultry.

**Table 1:** Influence of bacteriophage therapy on pathogenic microbial load in the gastrointestinal tract and internal organs of poultry.

Targeted Bacteria	Phage	Dose and Route	Main Outcomes	References
*Campylobacter*	**Phage Mixture** *Fletchervirus* phage NCTC 12673 + *Firehammervirus* phage vB_CcM-LmqsCPL1/1	Phage Mixture: 8.94 × 10^6^ PFU per bird via drinking water	Using a phage mixture reduced Campylobacter load in fecal samples by up to 1.1 log_10_ CFU/mL compared to the infected control	[64]
*Salmonella* *(S. typhimurium* and *S. enteritidis)*	BP cocktail (Belonging to the Myoviridae family)	10^6^ PFU/g of feed	At 7, 14, and 21 days, the application of the BP cocktail showed a significant reduction in *Salmonella* colonization in the broilers’ liver, crop, spleen, and caeca	[65]
*E. coli*, *Clostridium perfringens*	*E. coli* phage cocktail	**Doses** 1 g/kg, and 2 g/kg via feed **Concentration** 10^10^ PFU/g	*C. perfringens* in the ileum decreased (*p* < 0.05) by adding 1 g/kg phage cocktail and 1 g/kg probiotic	[66]
*Salmonella enteritidis*	Bacteriophage cocktail/BC containing G3D03, L1R06, and L1N01	10^9^ PFU/mL oral administration	In comparison to the infected control group, the data showed that BC supplementation lowered bacterial concentrations in the liver, spleen, heart, and cecum	[67]
*Salmonella enteritidis*	Commercial BP (ProBe-Bac^®^)	1 g/kg and 1.5 g/kg via diet	At 7- and 14-day post-challenge, BP supplementation significantly (*p* < 0.05) decreased *S. enteritidis* and coliform bacteria count in the cecum of chickens in comparison to the infected untreated group	[68]
*Salmonella* *enteritidis*	UPWr_S134 phage cocktail	**Dose** 3 × 10^10^ PFU/mL inoculated orally **Concentration** 1 × 10^7^ PFU/mL	Phage treatment dramatically reduced the number of *S. enteritidis* in internal organs (such as the liver, spleen, and cecal tonsils) compared to the infected untreated group	[69]
*Salmonella* *typhimurium*	Phage cocktail (SPFM10 and SPFM14)	**Three doses** 1 × 10^5^, 0.1 × 10^6^, and 10 × 10^7^ PFU/kg/day/chicken via feed **Concentration** 3 × 10^11^ PFU/L	Phage treatment significantly (*p* < 0.05) decreased *Salmonella* colonization and its counts in feces compared to the infected untreated group	[70]
*Proteobacteria* (*E. coli*, *Campylobacter jejuni*, *Salmonella typhimurium*)	Bacteriophage Cocktail (Bacter Phage C) (CTCBIO Inc., Seoul, Korea)	500 ppm via diet	BP supplementation in combination with β-mannanases decreased *Proteobacteria* and increased *Bacteroidetes* in the cecum	[71]
*Clostridium perfringens*	Powdered and encapsulated BP	10^6^ PFU/g of diet	Dietary BP supplementation significantly (*p* < 0.05) decreased cecal *C. perfringens* counts compared to the infected control group	[72]
*Escherichia coli*	*E. coli* O78 bacteriophage	10^8^ PFU/mL intratracheally	When compared to the infected non-treated group, BP treatment resulted in a significantly lower *E. coli* numbers in the lungs	[73]
*Salmonella*	Phage cocktail (SK-E1, SK-Ti1 SK-T2)	1 mL of 10^8^ PFU/mL (drinkers) and 1 mL of 10^7^ PFU/mL (Shavings)	*Salmonella* counts in drinking water were lowered by the phage cocktail by up to 2.80 log10 units, and in shavings, by up to 2.30 log10 units	[74]
*S. enteritidis*	Phage cocktail, UPWr_S134,	1 × 10^7^ PFU/mL via drinking water	In experimentally challenged chickens, the phage cocktail significantly reduced the count of *Salmonella*	[75]
*Salmonella* spp. *E. coli*; *C. perfringens*	Commercial Bacteriophage Product (CJ Cheiljedang Corp; Seoul, South Korea) contained mixture of phages targeting *Salmonella* spp. *E. coli;* *C. perfringens*	0.5 g/kg, 1.0 g/kg via feed **Concentration** 1 × 10^8^ PFU/g (For each of the *Salmonella* and *E. coli*) and 1 × 10^6^ PFU/g (For *C. perfringens*)	BP Supplementation increased *Lactobacillus* count in excreta and ileum compared to the group supplemented with antibiotics, while *Salmonella* and *C. perfringens* numbers were comparable in BP and antibiotic-supplemented groups	[76]
*Campylobacter jejuni*	Φ16-izsam Φ7-izsam	**Φ16-izsam** 10^8^ PFU/mL via oral gavage **Φ7-izsam** 10^7^ PFU/mL, via oral gavage	In comparison to the infected unsupplemented group, the BP supplementation of Φ16-izsam and Φ16-izsam significantly decreased *Campylobacter* counts to 1 log_10_ CFU/g and 2 log_10_ CFU/g, respectively, in the cecum	[77]
*Clostridium perfringens*	φCJ22	10^5^, 10^6^, and 10^7^ PFU/kg of diet	Phage inclusion at 10^6^ and 10^7^ decreased (*p* < 0.05) *C. perfringens* counts in the cecum (up to 1.24 log), relative to the challenged control group	[78]
*Clostridium perfringens*	Podovirus *C. perfringens* phage	**Dose** 0.5 mL via oral gavage **Concentration** 10^9^ PFU/mL	Phage treatment reduced the cecal *C. perfringens* counts compared to the infected control group	[79]
*E. coli*, *Salmonella*	Bacteriophage cocktail (*S. gallinarum*, *S. typhimurium*, *S. enteritidis*)	**Doses** 0.25 g BP/Kg, 0.5 gBP/Kg via feed **Concentration** 10^8^ PFU/g	The supplementation of BP significantly (*p* < 0.0001) decreased cecal *E*. *coli* and *Salmonella* counts compared with the control group fed only a basal diet	[80]
*E. coli* (Strain E28)	Phage cocktail 1-**Six-phage trial** [Phages AB27, TB49, G28, TriM, KRA2, and EW2] 2-**Four-phage trial** [Phages AB27, TB49, G28, and EW2]	log10 4.6 PFU/mL (Six phage) log10 6.7 PFU/mL (four phage) drinking water	In comparison to the infected control group, the number of *E. coli* bacteria in the feces of birds supplemented with four and six phages was reduced (*p* < 0.0001) and demonstrated a 0.7 log unit drop	[81]
*Campylobacter* (*C. jejuni* NCTC 12662, *C. jejuni* NC3142, and *C. coli* NC2934)	Phage cocktail (4 phage cocktail consisting of PH5, PH8, PH11, PH13 and 2 Phage Cocktail consisting of PH18, PH19)	3 mL of 10^7^ PFU/mL via drinking water	In comparison to the non-supplemented control group, phage administration significantly decreased the amount of *Campylobacter* in the ceca (range 1–3 log_10_ CFU/g)	[82]
*Salmonella enteritidis*	Bacteriophage from CTCBIO Inc., Seoul, Republic of Korea, consisting of a lytic bacteriophage specifically targeting *S. enteritidis* (KCTC 12012BP)	**Doses** 1 kg BP/metric ton 1.5 kg BP/metric ton Via feed **Concentration** 10^8^ PFU/g	Both BP treatments decreased (*p* < 0.001) the number of *S. enteritidis* in the ceca and cloacal swabs	[83]
*Salmonella* Kentucky and *E. coli*	*S.* Kentucky and *E. coli* O119 bacteriophages	***S.* Kentucky BP** 0.1 mL orally Concentration 10^8^ PFU/mL ***E. coli* O119 BP** Dose 0.1 mL orally Concentration 10^2^ PFU/mL	Phage therapy significantly (*p* < 0.05) decreased *S.* Kentucky and *E. coli* O119 levels in the cecum and liver when compared to the infected control group	[84]
*Salmonella* *typhimurium*	STP4-a	10^9^ PFU/g via feed	Pre-administration of phage STP4-a in the feed resulted in undetectable (*p* < 0.05) *Salmonella* numbers in feces compared to the infected control group	[85]
*Salmonella* (non-typhoid)	Phage cocktail (SalmoFREE^®^)	1 *×* 10^8^ PFU/mL in drinking water	Phage treatment reduced *Salmonella* incidence up to 100% compared to the untreated control group	[86]
*E. coli* (APEC O78)	APEC O78-specific bacteriophage	10^8^ PFU (Intratracheal inoculation)	In comparison to the untreated, uninfected control group, bacteriophage treatment significantly decreased *E. coli* shedding in the infected group	[87]
*S. enteritidis*	Lytic Bacteriophages (LBs)	10^9^ PFU/mL via drinking water	Phage therapy showed a significant reduction of up to 1.08 log_10_ CFU/g in the average number of intestinal *S. enteritidis* compared with the infected control group	[88]
*S. typhimurium*, *S. enteritidis*	Bacteriophage (Specific Lytic Phage against *S. typhimurium*, and *S. enteritidis*)	**1-*S. typhimurium* BP/chick** 0.1 mL orally Concentration 1.18 × 10^11^ PFU/mL **2-*S. enteritidis* BP/chick** 0.1 mL orally Concentration 1.03 × 10^12^ PFU/mL	Infected birds treated with bacteriophage resulted in no *Salmonella* colonization in the caecum at the end of the experiment	[89]
*S. enteritidis*	Bacteriophage commercial product containing 2 bacteriophages SP-1 and STP-1	0.1% BP and 0.2% BP via diet	Supplementation of BP at 0.2% significantly (*p* < 0.05) reduced nalidixic acid-resistant *S. enteritidis* numbers in the caecum, spleen, ovary, and feces compared to the infected control	[90]
Avian pathogenic *Escherichia coli* (APEC)	**Naked phage** [ΦKAZ14], and Chitosan nanoparticles loaded phage [C-ΦKAZ14 NPs]	**Dose** 0.2 mL orally **Concentration** 10^7^ PFU/mL	Chitosan nanoparticles loaded bacteriophage (ΦKAZ14) treatment significantly reduced *E. coli* count in feces, lungs, and spleen compared to the naked phage ΦKAZ14-treated group and the untreated infected control	[91]
*S. enteritidis*	PSE phage	10^6^ PFU/mL via oral gavage	BP administration significantly increased lactic acid bacteria count and decreased colibacilli and total aerobes count in the ileum compared to infected and uninfected controls. Prophylactic administration of BP reduced *S. enteritidis* more effectively in cecal tonsils compared to infected and therapeutic phage-supplemented groups (20% vs. 100%)	[92]
*Salmonella* spp.	Commercial Bacteriophage product (From CTCBIO Inc., Seoul, Republic of Korea) containing a mixture of BP designed to lyse several key *Salmonella* serovars and *S. aureus*	**Doses** 0.4 g/kg BP 0.8 g/kg BP via feed **Concentration** 10^8^ PFU per g	BP supplementation significantly decreased caecal *Salmonella* species compared to the group fed a basal diet only	[93]
*Salmonella* spp. and *Clostridium perfringens*	Commercial Bacteriophage product (From CTCBIO Inc., Seoul, Republic of Korea) containing a mixture of individual BP targeting *Salmonella* spp. and *C. perfringens*	**Dose** 0.5 g/kg via diet **Concentration** 10^8^ PFU per g and 10^6^ PFU per g	BP addition in the diet lowered (*p* < 0.05) DNA copy numbers of *C. perfringens* compared to the negative control group who was fed a basal diet only	[94]
*Salmonella enteritidis*	Bacteriophage P22	10^9^ PFU/mL via oral gavage	Broilers challenged with *Salmonella enteritidis* and treated with P22 reduced the *Salmonella enteritidis* counts in the caeca and crop to less than the detection limit of 10^2^ CFU/g	[95]
*Campylobacter* *jejuni*	Phage cocktail (Phages 1, 2, 5, and 13 from British *Campylobacter* phage typing scheme)	10^7^ PFU per ml via crop	Treatment with phage cocktail and single phage significantly reduced *Campylobacter* load in the caecum up to log_10_ 2.8 CFU/g	[96]
*E. coli* and *Salmonella*	Bacteriophage containing *S. gallinarum*, *S. typhimurium*, and *S. enteritidis* at the ratio of 3:3:4.	**Doses** 0.25 g/kg feed 0.5 g/kg feed **Concentration** 10^8^ PFU per gram	BP supplementation significantly (*p* < 0.05) reduced *E. coli* and *Salmonella* counts in excreta while increasing *Lactobacillus* counts compared to the negative control group fed only a basal diet and the antibiotic-supplemented positive control group, respectively	[97]
*Salmonella enteritidis*	Anti-SE bacteriophage	**Doses** 0.05, 0.1, and 0.2% via feed **Concentration** 10^9^ PFU/g	Anti-SE bacteriophage supplementation significantly reduced *S. enteritidis* concentration in the caecum in comparison to the control group fed only a basal diet	[98]
*Salmonella enteritidis*	Bacteriophage ΦCJ07	10^9^ PFU/g 10^7^ PFU/g 10^5^ PFU/g via feed	BP supplementation at 10^9^ PFU/g and 10^7^ PFU/g showed significantly (*p* < 0.05) lower mean intestinal *S. enteritidis* counts in the challenged and contact birds compared to the infected untreated control	[99]
*Salmonella* spp. and *E. coli*	Bacteriophages used (*S. gallinarum* BP, *S. typhimurium* BP, and *S*. *enteritidis* BP)	**Doses** 0.020% 0.035% 0.050% via feed **Concentration** 10^8^ PFU per gram	In comparison to the control group that was given only basal feed, bacteriophage supplementation significantly decreased the concentrations of *Salmonella* spp. and *E. coli* in the excreta	[100]
*S. typhimurium*	Phage cocktail (UAB_Phi20, UAB_Phi78, and UAB_Phi87)	10^11^ PFU/mL via oral gavage	Birds challenged with *S. typhimurium* and treated with phage cocktail showed reductions (*p* < 0.0001) in the *Salmonella* concentrations in the cecum by days 2, 6, and 8 postinfection (4.4 log_10_, 3.2 log_10_, and 2 log_10_, respectively), and at the end of the experiment	[101]
*Salmonella gallinarum*	Bacteriophage CJø01	10^6^ PFU/kg via feed	Bacteriophage supplementation in contact birds (birds placed in the same cage with orally challenged *S. gallinarum* birds) decreased *S. gallinarum* invasion in the liver, spleen, and caecum compared to the untreated contact birds	[102]
*Campylobacter jejuni* *Campylobacter coli*	Phage CP220	The phage doses were 5, 7, and 9 log PFU administered in 1 mL of 30% (wt/vol) CaCO_3_ by oral gavage	When compared to the uninfected and infected controls, treatment with 7 and 9 log PFU of phage CP220 significantly reduced the mean cecal counts of *C. jejuni* and *C. coli*, respectively	[103]
*Salmonella enteritidis*	Bacteriophage cocktail (BP1, BP2, and BP3)	10^8^ PFU/mL administered via coarse spray and drinking water	Chickens challenged with *S. enteritidis* and treated with BP showed significantly lower intestinal *S. enteritidis* numbers than the infected control	[104]
*Salmonella* *(S. enteritidis*, and *S*. *typhimurium)*	Three bacteriophages (Φ151, Φ25, Φ10)	**Lower Phage Titer** 1 mL of 9 log10 PFU/mL **Higher Phage Titer** 1 mL of 11.0 log10 PFU/mL via oral gavage	Broilers challenged with *Salmonella* and treated with a high phage titer showed a significant reduction in cecal colonization by *S. enterica* serotypes Enteritidis and Typhimurium compared to the infected control	[105]
*Salmonella enteritidis*	Phage cocktail (CB4φ, wt45φ)	10^8^ PFU/mL chick via oral gavage	After 24 h, birds challenged with SE and treated with a phage cocktail had significantly lower SE load in cecal tonsils than the infected control	[106]
*Campylobacter jejuni* (HPC5 and GIIC8)	Bacteriophages (CP8 and CP34)	**Doses** log10 5, 7, and 9 PFU were administered in 1 mL of 30% (wt/vol) CaCO_3_ via oral gavage	Treatment of *C. jejuni* HPC5-colonized chickens with phage CP34 at varying dosages led to a significant reduction in intestinal *Campylobacter* counts compared to the untreated control. Similarly, birds infected with *C. jejuni* GIIC8 and treated with phage CP8 at a dose of log_10_ 7 PFU had significantly lower cecal *Campylobacter* counts	[107]
*Campylobacter jejuni*	Phage 71 (NCTC 12671) and Phage 69 (NCTC 12669)	**Phage 71 doses in PFU/mL** 4 × 10^11^ 2 × 10^10^ 5 × 10^10^ 4 × 10^10^ oral gavage **Phage 69 doses in PFU/mL** 3 × 10^10^ 5 × 10^10^ 2 × 10^10^ 2 × 10^10^	Birds challenged with *C. jejuni* and given phage 71 and 69 (4-day post-treatment) decreased *C. jejuni* colonization in the caecum by 1 log_10_ CFU/g than the infected control	[108]
*Salmonella typhimurium*	Phage cocktail (S2a, S9, and S11)	**Dose** 0.5 mL/bird orally **Concentration** 5.4 × 10^6^ PFU	Broilers challenged with ST and treated with phage cocktail showed significantly lower ST counts in the ileum compared to the infected control group (1.1 CFU/mL vs. 81.8 CFU/mL)	[109]

SPF, specific pathogen free; SE, *Salmonella* Enteritidis; ST, *Salmonella* Typhimurium; BP, Bacteriophage; BC, Bacteriophage cocktail.

#### 4.2.2. Effects on Production Parameters of Poultry

Table 2 presents the effects of bacteriophage application on key production indices in poultry, including improvements in body weight gain, FCR, and livability. The findings also indicate that bacteriophages effectively regulate major enteric bacteria, positioning them as a viable alternative to conventional antibiotics.

**Table 2:** Impact of bacteriophage use against major enteric bacteria on poultry production parameters.

Targeted Bacteria	Phage	Dose and Route	Main Outcomes	References
*E. coli, Clostridium perfringens*	*E. coli* phage cocktail	**Dose** 1 g/kg, and 2 g/kg feed **Concentration** 10^10^ PFU/g	Supplementation of phage cocktail and probiotic, both alone or in combination, significantly improved FCR, relative thymus weight, and relative heart weight compared to the infected control group	[66]
*Salmonella infantis*	Autophages phages	10^8^ PFU/mL via spray	Phage application reduced *Salmonella* positivity from 100% to 36% in the flock	[110]
*Salmonella*	*Salmonella*-specific phage cocktail (SPC) SP 75 SP 100 SP 175	0.075 g/kg, 0.1 g/kg and 0.175 g/kg via feed	The addition of SPC in the feed significantly (*p* < 0.05) improved FI and breast weight when compared to the negative control group fed only the basal diet	[111]
*Salmonella enteritidis*	Bacteriophage cocktail (BC) containing G3D03, L1R06, and L1N01	10^9^ PFU/mL oral administration	Supplementing BC decreased the mortality rate and improved the BWG of chicks compared to the infected control group	[67]
Avian pathogenic *Escherichia coli*	*E. coli* phage CE1	0.1 mL of 10^8^ PFU/mL Intramuscular injection	Treatment with phage resulted in no mortality in the challenged chickens and was found to be effective in treating colibacillosis	[112]
*Salmonella* spp. (Nontyphoid)	Phage cocktail SPFM10 SPFM14	**Three doses** 1 × 10^5^, 0.1 × 10^6^, and 10 × 10^7^ PFU/kg/day/chicken via feed **Concentration** 3 × 10^11^ PFU/L	Phage treatment at all three doses in the challenged birds increased BWG, FI, and decreased mortality % in comparison to the challenged birds with no phage in their diet	[70]
Nalidixic acid-resistant *Salmonella enteritidis*	Commercial BP Product from CTCBIO Inc., Seoul, Republic of Korea, consisting of a lytic bacteriophage specifically targeting *S. enteritidis* (KCTC 12012BP)	10^8^ PFU/g via diet	BP supplementation significantly improved adjusted FCR compared to the infected control group	[83]
*Clostridium perfringens*	Powdered and encapsulated BP	10^6^ PFU/g of diet	The addition of BP to the diet improved FI, BWG, and FCR compared to the non-supplemented group, and BP-fed groups showed the highest cecal short-chain fatty acids compared to uninfected and infected control groups	[72]
*Escherichia coli*	*E. coli* O78 bacteriophage	10^8^ PFU intratracheally	The bacteriophage application in the challenged group significantly increased BW, BWG, and improved FCR compared to the infected–antibiotic-treated group	[73]
*Salmonella* spp; *E. coli*; *Clostridium perfringens*	Commercial BP Product (CJ Cheiljedang Corp; Seoul, South Korea) contained mixture of phages targeting *Salmonella* spp. *E. coli*; *C. perfringens*	0.5 g/kg and 1.0 g/kg Via feed **Concentration** 1 × 10^8^ PFU/g (For each of the *Salmonella and E. coli*) and 1 × 10^6^ PFU/g (For *C. perfringens*)	Throughout the experiment, the BP supplementation significantly increased BWG in comparison to the control group who was fed a basal diet	[76]
*Clostridium perfringens*	Podovirus *C. perfringens* phage	**Dose** 0.5 mL via oral gavage **Concentration** 10^9^ PFU/ml	Phage treatment led to a significant reduction in mortality relative to the infected control	[79]
*Clostridium perfringens*	φCJ22	10^5^, 10^6^, and 10^7^ PFU/kg of diet	Compensated FI, BWG, and FCR relative to the challenged control after disease challenge Decreased mortality rates (*p* < 0.05)	[78]
*E. coli*; *Salmonella*	Bacteriophage cocktail (*S*. *gallinarum*, *S*. *typhimurium*, *S*. *enteritidis*)	**Doses** 0.25 g BP/Kg, 0.5 g BP/Kg via feed **Concentration** 10^8^ PFU/g	BP used at 0.5 g/kg significantly increased BWG compared to the control group fed only a basal diet	[80]
*Salmonella* and *E. coli*	*S*. *Kentucky* and *E. coli* O119 bacteriophages	***S*. Kentucky BP** 0.1 mL orally Concentration 10^8^ PFU/mL ***E. coli* O119 BP** 0.1 mL orally Concentration 10^2^ PFU/mL	Broilers challenged with *S*. Kentucky and *E. coli* O119 and treated with phage showed no mortality compared to the infected control (30% mortality)	[84]
*E. coli* (O78:K80, O2:K1)	**Single phage** (TM3) **Phage cocktail** (TM1, TM2, TM3, TM4, TM5)	10^10^ PFU in 200 μL via intramuscular injection	On days 7, 14, and 21 post-challenge, birds treated with the phage cocktail had a greater BW (*p* < 0.05) compared to infected control Moreover, the birds challenged with *E. coli* and treated with either a single phage or phage cocktail showed a significant decrease in mortality (26.3% and 13.3%, respectively) compared to the untreated infected control (46.6%)	[113]
*E. coli* (APEC O78)	APEC O78-specific bacteriophage	10^8^ PFU (Intratracheal inoculation)	Bacteriophage treatment reduced the mortality associated with infection of *E. coli*	[87]
Avian pathogenic *E. coli*	**Naked phage** (ΦKAZ14); **Chitosan nanoparticles loaded phage** (C-ΦKAZ14 NPs)	**Dose** 0.2 mL orally **Concentration** 10^7^ PFU/mL	Chitosan nanoparticles loaded bacteriophage (ΦKAZ14) treatment significantly improved BW and decreased mortality % compared to the infected untreated group	[91]
*Salmonella* spp; *C. perfringens*	Commercial BP Product (From CTCBIO Inc., Seoul, Republic of Korea) containing a mixture of individual BP targeting *Salmonella* spp. and *Clostridium* perfringens	**Dose** 0.5 g/kg via diet **Concentration** 10^8^ PFU per g 10^6^ PFU per g	BP addition to the diet resulted in better FCR (*p* < 0.05) relative to the negative control treatment, fed only the basal diet	[94]
*E. coli*	SPR02	**Dose** 200 mL sprayed on the surface area of 3.9 m^2^ pens **Concentration** 10^8^ PFU/ml	Bacteriophage treatment of the litter significantly reduced mortality in the challenged birds	[114]
*E. coli*	Bacteriophage SPR02	1 ml of 3.9 × 10^9^ pfu (via spray); and 0.1 ml of 3.9 × 10^8^ pfu per bird (Intratracheal)	Intratracheal administration of BP SPR02 in challenged birds significantly decreased mortality % compared to the untreated challenged treatment	[115]
*Salmonella* *enteritidis*	Anti-SE bacteriophage	0.05, 0.1 and 0.2% via feed **Concentration** 10^9^ PFU/g	Anti-SE bacteriophage supplementation at 0.2% significantly decreased mortality % and increased relative leg muscle weight compared to the infected unsupplemented group	[98]
*Salmonella* spp.; *E. coli*	Bacteriophages used (*S. gallinarum* BP, *S. typhimurium* BP and *S. enteritidis* BP)	**Doses** 0.020% 0.035% 0.050% via feed **Concentration** 10^8^ PFU per gram	Bacteriophage supplementation at all three doses resulted in a significant increase in egg production in comparison to the control group that fed only a basal diet during weeks 4 to 6. Moreover, BP at inclusion levels of 0.020% and 0.050% showed greater (*p* < 0.05) Haugh unit (HU) relative to the control group fed only a basal diet during weeks 5 and 6	[100]
*Salmonella gallinarum*	Bacteriophage CJø01	10^6^ PFU/kg via feed	Bacteriophage supplementation significantly decreased mortality in contact birds compared to unsupplemented contact birds	[102]
*E. coli*	**Experiment-1** phi F78E **Experiment-2** Phage cocktail (phi F78E, phi F258E and phi F61E)	**phi F78E** Doses 1 mL orally 1 mL spray Concentration 1.5 × 10^9^ PFU/mL **Phage cocktail** 5.0 × 10^7^ PFU/mL via spray and orally	Bacteriophage administered at 1.5 × 10^9^ PFU/mL significantly decreased mortality and morbidity compared to the untreated infected group. In the second experiment, a phage cocktail administered at an inclusion level of 5.0 × 10^7^ PFU/mL reduced flock mortality (to <0.5%) in *E. coli-infected* flock	[116]
*E. coli*	Bacteriophage EC1	0.2 mL of bacteriophage EC1 (10^11^ PFU/mL) via intratracheal	Infected birds treated with bacteriophage EC1 had higher body weight (15.4%) and lower mortality rates compared to untreated birds (13.3% vs. 83.3%)	[117]
*Clostridium perfringens*	Phage cocktail (INT-401)	0.5 mL (2.5 × 10^9^ PFU/bird via oral gavage	Birds challenged with CP and treated with INT-401 significantly (*p* < 0.05) reduced mortality (by 92%) and improved BWG and FCR compared to untreated infected controls	[118]
*E. coli*	Two bacteriophages (SPR02 and DAF6 used separately)	**Dose** 0.1 mL via IM injection **Concentrations** 4 × 10^9^, 10^7^, 10^5^, or 10^3^ PFU/ml	Broilers infected with *E. coli* and treated with BP SPR02 at a phage titer of 10^8^ exhibited significantly decreased mortality compared to the infected group (7% vs. 48%)	[119]
*Salmonella typhimurium* (ST)	Phage cocktail (S2a, S9, and S11)	5.4 × 10^6^ PFU/0.5 mL/bird via orally	Broilers challenged with ST and treated with phage cocktail resulted in a significantly higher BWG compared to the infected control	[109]
*E. coli*	Phage cocktail (DAF6 and SPR02)	3.7 × 10^9^ and 9.3 × 10^9^ PFU/mL, DAF6 and SPR02, respectively, via injection in the thigh	Birds challenged with *E. coli* and treated with BP cocktail significantly decreased mortality % compared to the infected control (15% vs. 68%)	[120]
*E. coli*	SPR02	**Study 1** 10^3^, 10^4^, 10^6^, and 10^8^ PFU via air sac inoculation **Study 2** 10^3^_,_ 10^4^_,_ 10^6^ PFU bacteriophage via drinking water	**Study1** Birds infected with *E. coli*, and treated with 10^3^, 10^4^, and 10^6^ PFU BP SPR02, significantly reduced mortality rates as compared to the infected control **Study 2** Birds challenged with *E. coli* and supplemented with phage SPR02 at the inclusion of 10^3^ PFU in drinking water resulted in a significantly higher BW compared to the infected control	[121]
*E. coli*	DAF6 and SPRO2	3.6 × 10^7^ and 4.6 × 10^7^ PFU/mL, of DAF6 and SPRO2, respectively (Aerosol spray)	Significantly improved BW and decreased mortality rate (50% less) compared to the PBS-treated challenged group on day 7	[121]
*Salmonella* spp.	3 phages 3ent1, 8sent65 and 8sent1748	**Dose** 1 L of BAFASAL^®^ for 75,000 birds **Concentration** 10^8^ PFU/mL via drinking water	Bafasel^®^ application reduced mortality and *Salmonella*-positive birds (by 94.2%), as well as improved FCR compared to the non-treated controls	[122]
*Salmonella* spp.	SalmoFree^®^ Sciphage	10^8^ PFU/mL via drinking water	SalmoFree^®^ eliminated the occurrence of *Salmonella* and had no impact on production metrics such as FI, BWG, and FCR	[86]
*Salmonella* spp.	Biotector S1^®^	**Broilers** 5 × 10^7^, 1 × 10^8^ and 2 × 10^8^ PFU/kg via feed **Broilers breeders** 1 × 10^6^ PFU/kg via feed **Layers** 1 × 10^8^ PFU/kg via feed	Phage application decreased mortality by 73% and 53% in challenged broilers and broiler breeders, respectively, compared to the infected control In layers, phage application at 1 × 10^8^ PFU/kg improved egg production by 3% (90.6% vs. 87.5% in control) and egg mass by 2.4% (59.2% vs. 56.8% in control)	[123]

FI, Feed intake; BWG, Body weight gain; FCR, feed conversion ratio; CP, *Clostridium perfringens*.

#### 4.2.3. Impact of Bacteriophages on Blood Constituents and Immune Response

Bacteriophage treatment has shown considerable potential in managing blood disorders and enhancing immunological responses in poultry. A phage cocktail administered at 1 g/kg significantly reduced cholesterol levels in male broiler chickens compared with the control group. Dlamini et al. [111] reported that a *Salmonella*-specific phage cocktail produced linear increases in monocytes, albumin, and globulin across different dosage levels in male broilers. In addition, Sarrami et al. [68] found that administering a commercial phage product (ProBe-Bac^®^) to broilers challenged with *Salmonella enteritidis* reduced serum aspartate aminotransferase levels and increased albumin, triglycerides, and the albumin-to-globulin ratio. Lee et al. [72] further demonstrated that supplementation with encapsulated bacteriophages in broilers infected with *C. perfringens* significantly elevated serum IgA concentrations. However, in contrast to these findings, Noor [80] reported that a phage cocktail had no measurable effect on the hematological parameters of broiler chickens (Table 3).

**Table 3:** Impact of bacteriophage therapy on blood constituents and the immune response of poultry challenged with enteric pathogens.

Targeted Bacteria	Animal/Model	Phage	Dose and Route	Main Outcomes	References
*E. coli*, *Clostridium perfringens*	Male Broilers (Cobb 500)	*E. coli* phage cocktail	1 g/kg, and 2 g/kg feed	Supplementation of 1 g/kg phage cocktail alone significantly decreased the cholesterol levels compared to the control group (0 g/kg) and the higher dosage of phage cocktail (2 g/kg)	[66]
*Salmonella*	Male Broiler chickens (Ross-308)	*Salmonella*-specific phage cocktail (SPC) SP 75 SP 100 SP 175	0.075 g/kg, 0.1 g/kg, and 0.175 g/kg via feed	Supplementation of SPC resulted in a linear increase in monocytes, albumin, and globulin	[111]
*Salmonella* *enteritidis*	As hatched broiler chickens (Ross-308)	Commercial BP (ProBe-Bac^®^)	1 g/kg 1.5 g/kg via diet	Supplementation of BP significantly decreased serum concentration of AST and increased concentrations of albumin, A/G ratio, and triglycerides	[68]
*Clostridium perfringens*	1-day-old broiler chickens (Ross-308 unsexed)	Powdered and encapsulated BP	10^6^ PFU/g of diet	Chickens that were fed a diet supplemented with encapsulated BP showed the most elevated (*p* < 0.05) serum IgA levels	[72]
*E. coli*, *Salmonella*	Broilers (day-old chickens)	Bacteriophage cocktail (S. *gallinarum*, S. *typhimurium*, S. *enteritidis*)	**Dose** 0.25 g BP/Kg, 0.5 g BP/Kg **Concentration** 10^8^ PFU/g	Bacteriophage supplementation did not significantly affect the blood profile (RBCs, PCV, Hb, WBCs, and lymphocytes) of experimental broiler chickens	[80]
*Salmonella* *enteritidis*	Single comb white leghorns (40 wk. old)	Commercial bacteriophage product containing 2 bacteriophages SP-1 and STP-1	0.1% BP and 0.2% BP via diet	Supplementation of BP at 0.1% and 0.2% significantly reduced IL-6 mRNA expression as compared to the infected control	[90]
*Salmonella* *enteritidis*	Male broilers (Ross 308)	Anti-SE bacteriophage	0.05%, 0.1% and 0.2% via feed **Concentration** 10^9^ PFU/g	Anti-SE bacteriophage supplementation decreased AST levels in blood and decreased LDL concentration in serum compared to the control group fed only a basal diet	[98]

RBCs, red blood cells; PCV, packed cell volume; Hb, hemoglobin; WBCs, white blood cells; AST, aspartate aminotransferase; LDL, Low-density lipoprotein; A/G, Albumin to globulin.

#### 4.2.4. Application of Phages in Postharvest Products (Biocontrol Agents)

Decontamination is a complicated process that comprises eliminating and inactivating the bacteria present in food items, hence improving their shelf life. The practice of using phages to reduce foodborne illness has been focused on decontaminating carcasses, raw meat, and ready-to-eat (RTE) items. Phage biocontrol is an innovative method that employs lytic phages to diminish the presence of pathogenic bacteria in food and meat products. Bacteriophages have demonstrated notable efficacy in reducing foodborne pathogens in poultry meat, with significant reductions observed in bacterial loads of *Salmonella* and *Campylobacter*. Several findings highlight the potential of phage therapy to reduce food safety concerns in poultry products associated with foodborne zoonotic pathogens (Table 4). Phage use is gradually becoming more widely recognized in the prevention of foodborne illnesses since the use of phage preparations for food applications was authorized. Furthermore, lytic phages have numerous benefits as biocontrol agents, and prior research has described the rationale for choosing this strategy over alternative options. Undoubtedly, there has been increased interest in phage biocontrol, but many challenges limit its application [55,124]. The titers of the phage used, the degree of pathogen contamination, the emergence of mutations resistant to phages, and the stability of phages on food products are some of the critical variables that affect the effectiveness of phage biocontrol.

Decontamination is a complex process involving the removal and inactivation of bacteria present in food products to enhance their shelf life. The use of bacteriophages to reduce foodborne illnesses has primarily focused on decontaminating carcasses, raw meat, and ready-to-eat (RTE) products. Phage biocontrol is an innovative approach that employs lytic phages to reduce pathogenic bacteria in food and meat products. Bacteriophages have shown substantial efficacy in lowering foodborne pathogens in poultry meat, with marked reductions in *Salmonella* and *Campylobacter* loads. Numerous studies underscore the potential of phage therapy to mitigate food safety risks associated with zoonotic pathogens in poultry products (Table 4). The use of phage-based preparations in food applications has gained increasing acceptance following regulatory authorization. Lytic phages offer several advantages as biocontrol agents, and previous research has outlined the rationale for selecting this strategy over alternative methods. Although interest in phage biocontrol continues to grow, several challenges still limit its broader implementation [55,124]. Key factors influencing the effectiveness of phage-based treatments include phage titer, the level of pathogen contamination, the emergence of phage-resistant bacterial mutants, and phage stability on food surfaces.

**Table 4:** Effects of bacteriophage application against foodborne zoonotic bacteria on postharvest poultry meat and meat products.

Targeted Bacteria	Animal/Model	Phage Product	Dose & Route	Main Outcomes	References
*S. enteritidis*	Chicken and turkey meat cuts	Commercial product (PhageGuard S, formerly called Salmonelex)	1 and 2 × 10^7^ PFU/cm^2^ The phage was applied by emersion to a final conc. of ∼7 log_10_ PFU/cm^2^	Inoculating chicken and turkey meat with 4 log10 CFU/cm^2^ of *S. enteritidis*, followed by PhageGuard S treatment at both doses, resulted in a reduction of more than 1 log_10_ CFU/cm^2^ in *S. enteritidis* after 24 h	[125]
*Salmonella* spp. from ground chickens and other sources	Postharvest *Salmonella*-free boneless, skinless chicken meat	Commercial phage (Salmonelex™)	(~10^7^ PFU/cm^2^) via tap and filtered water	When phage (diluted in sterile tap water) was applied to boneless chicken thighs and legs experimentally contaminated with *Salmonella* serovars, there was a more significant (*p* < 0.05) decrease in *Salmonella*, with a 0.39 log CFU/cm^2^ decrease observed, as opposed to a 0.23 log_10_ CFU/cm^2^ decrease with sterile filtered water	[126]
*Salmonella* spp.	Chicken breast	Commercial phage (SalmoFresh)	10^8^ and 10^9^ PFU/mL via spray (surface application) for the samples up to 7 days of storage at 4 °C	Applying phage at 9 log PFU/mL decreased *Salmonella* by 1.0–1.1 log_10_ CFU/g, whereas 8 log PFU/mL achieved a reduction of 0.5–0.6 log_10_ CFU/g	[127]
*Salmonella* *typhimurium*	Chicken breast and chicken mince	P22 phage	8 mL of P22 phage, having a concentration 10^12^ PFU/ml	In sliced breast and minced chicken, the P22 phage significantly decreased the *Salmonella* population by 1.0–2.0 log cycles	[128]
*S. typhimurium* and *S. enteritidis*	Chicken breasts	Bacteriophage Cocktail (UAB_Phi 20, UAB_Phi78, UAB_Phi87)	100 mL phage cocktail having a concentration 10^9^ PFU/mL	After dipping chicken breasts in a phage cocktail solution for five minutes and refrigerating at 4 °C for seven days, a significant decrease of 2.2 log_10_ CFU/g for *S.* typhimurium and 0.9 log_10_ CFU/g for *S. enteritidis* was recorded	[129]
*S. enteritidis* and *S. typhimurium*	Meat (chicken skin)	wksl3 phage	2.2 × 10^8^ PFU/mL via spray	*Salmonella* levels decreased by almost 2.5 logs after phage wksl3 was applied to chicken skin at 8 °C	[130]
S. *enteritidis*	Meat (chicken skin)	Phage cocktail	100 mL used, having a concentration 10^9^ PFU/mL via dipping	Bacteriophage cocktail application to chicken skin experimentally contaminated with *S. enteritidis* significantly decreased *S. enteritidis* by 1.0 log_10_ CFU/cm^2^ compared to the untreated infected control	[131]
*Campylobacter jejuni* NCTC 11168 and *Campylobacter coli* NCTC 12668	Meat (Raw chicken)	Phage CP81, and Phage NCTC12684	1 mL phage lysate applied to the meat stored at 4 °C for up to 1 week	No reduction in *Campylobacter* number was detected in chicken meat following phage treatment	[132]
*S. enteritidis*	Broiler Meat	PHL 4 (*Salmonella enteritidis* phage type 13A)	5.5 mL of 10^8^ or 10^10^ PFU/mL via spray	The broiler carcasses treated with 5.5 × 10^8^ or 10^10^ PFU of PHL 4 phage resulted in a 93% decrease in *S. enteritidis* levels as compared to the control group sprayed with sterile saline	[133]
*Campylobacter* *jejuni*	Meat (chicken skin)	*C. jejuni* phage 12673	**Dose** 10^6^ PFU/cm^2^ Applied to chicken skin **Concentration** log_10_ 9.2 and 10.1 PFU/ml	Treatment with phage at 10^6^ PFU/cm^2^ significantly lowered the *Campylobacter* numbers by almost 95%, compared to the untreated group	[134]
*S. enteritidis*	Meat (chicken skin)	Bacteriophage P22, 29C	**P22 phage** 7.1 × 10^2^ PFU/mL **29C phage** 8.1 × 10^2^ PFU/mL via spray	BP P22 and 29C were administered to chicken skin (experimentally infected with *S. enteritidis*), significantly (*p* < 0.01) decreasing S. *enteritidis* by 98.7 and 99.2%, respectively, as compared to the untreated infected control	[134]
*Campylobacter* *jejuni*	Meat (chicken skin)	Bacteriophage Φ2	10^7^ PFU	Phage application significantly (*p* < 0.0001) reduced *Campylobacter* recovery by 1log_10_ CFU/cm^2^ as compared to untreated inoculated control	[135]

## 5. Role of Bacteriophages as Disinfectants in Poultry Operations

Several strategies, such as stringent biosecurity measures, regulatory controls, and specific management practices, have been proposed to reduce bacterial contamination associated with poultry production. Products derived from bacteriophages can be applied as bio-sanitizers on food-contact surfaces, as well as in farms, hatcheries, transport crates, and poultry processing facilities. In addition, bacteriophages have proven effective in preventing the formation of biofilms and limiting the spread of mature biofilms produced by pathogenic microorganisms on surfaces commonly found in poultry operations [136].

Phage-based surface disinfectants targeting *Salmonella*, such as BacWash™ (OmniLytics Inc., Sandy, UT, USA), can be sprayed, misted, or applied as a wash to live birds before slaughter. Similarly, Ecolicide PX™ (Intralytix, Columbia, MD, USA), which contains *E. coli* O157:H7-specific phages, has been developed to decontaminate the skin of live animals before slaughter [123]. Treating litter with phage preparations targeting *E. coli* has also been shown to protect broiler chickens against colibacillosis [114]. Likewise, bacteriophage applications have demonstrated efficacy in reducing recoverable *Campylobacter jejuni* on experimentally contaminated broiler skin [135].

## 6. Emerging Trends in Phage Applications

Biosensor-based phage technologies have rapidly evolved beyond simple detection tools and now constitute a versatile platform for targeted therapeutic and vaccine delivery. Reporter phages, engineered by inserting genes encoding fluorescent, luminescent, or enzymatic markers, have already demonstrated strong utility in detecting foodborne pathogens such as *Salmonella* [137], *E. coli* [138], and *Listeria monocytogenes* (*L. monocytogenes*) [139]. Advances in phage engineering, supported by multi-omics technologies, continue to expand the analytical and diagnostic potential of these constructs.

A growing body of evidence shows that phages can be adapted as delivery vehicles for therapeutic compounds by encapsulating small molecules or by displaying functional peptides on their capsid surfaces. For example, M13 phage particles displaying epitopes of the influenza A virus have elicited strong humoral responses in murine models and demonstrated protection against viral challenge [140]. Similarly, T4 phage nanoparticles engineered to present plague or anthrax antigens have induced robust systemic and mucosal immunity, providing a stable and safe vaccine platform with high antigen payload capacity [141]. Phage display systems have also been exploited for cancer immunotherapy; M13-based vectors presenting tumor-associated antigens have shown promising antitumor responses in preclinical studies [142]. These developments are driven by advantageous intrinsic properties of phages, including structural uniformity, high stability, biodegradability, and the ability to be produced at low cost, which collectively position them as efficient scaffolds for virus-like particle vaccine design [143].

While these developments are well established in human biomedical research, equivalent applications in livestock remain limited. Current veterinary phage use is largely restricted to antibacterial interventions, and systematic studies on phage-mediated drug or vaccine delivery in poultry or other farm animals are only emerging. Given the rapid advancements in phage engineering and the growing need for alternatives to conventional antimicrobials, it is reasonable to expect that phage-based veterinary pharmaceuticals will gain traction in the coming years.

## 7. Pros and Cons of Bacteriophages

Bacteriophages possess several characteristics that make them strong candidates for replacing conventional antibiotics. Unlike broad-spectrum antibiotics, phages exhibit a high degree of host specificity, reducing the risk of gut dysbiosis and secondary infections. Moreover, developing a new antibiotic requires substantial time and resources, whereas isolating, propagating, and producing phages at scale is considerably more economical. Phages also offer additional benefits, including the ability to cross the blood–brain barrier, disseminate throughout the body following systemic administration, and inhibit biofilm formation [144,145]. Resistance dynamics further strengthen their therapeutic potential. Bacterial resistance to antibiotics presents a significantly greater clinical challenge than resistance to phage therapy. Modern genetic engineering enables the modification of phages to counteract emerging bacterial defenses, a strategy that does not apply to controlling antibiotic resistance. Notably, even bacteria that are resistant to all available antimicrobial treatments often remain susceptible to phage infection, provided that suitable natural or engineered phages are available.

Although bacteriophages offer many advantages, they also present several limitations. Preparing phages for therapeutic application is complex, and many unresolved challenges stem not from phage biology itself but from practical and regulatory considerations [146]. Their narrow host range often restricts their ability to target all pathogenic strains within a bacterial species or genus [147]. While a given phage may be effective against a specific pathogen, clinical infections are frequently polymicrobial. Consequently, using highly specific phages alone may fail to provide sufficient therapeutic coverage [147,148]. Additional challenges arise from the use of temperate (lysogenic) phages. Because lysogenic phages integrate into the host genome rather than immediately killing the bacterium, their use can render therapy ineffective. In animal prophylactic applications, phage preparations may also promote the emergence of mutant phages, as they do not eliminate phages immediately after application [123]. Furthermore, characteristics of the temperate phage cycle can increase the risk of disseminating harmful genes in the environment. Lysogenic phages can transfer toxins and antibiotic resistance genes to bacteria, posing a significant public health concern [149]. Current evidence indicates that phages must be applied at high concentrations to achieve meaningful reductions in foodborne infections and mortality [150,151]. However, using large doses or prolonged exposure to food products may trigger the production of neutralizing antibodies in humans. High-concentration phage use may also lead to phage accumulation in various body organs.

## 8. Challenges of Using Phages in Poultry and Potential Solutions

Despite growing interest in bacteriophage-based interventions for poultry, several scientific, technical, and regulatory obstacles hinder large-scale implementation. These challenges can be broadly categorized into issues related to bacterial resistance, optimal phage selection, delivery and stability within the host, regulatory approval, ecological impacts, and variability in in vivo pharmacokinetics [152].

### 8.1. Development and Risk of Phage Resistance

Bacteria employ diverse strategies to evade bacteriophage predation, creating a significant challenge for phage-based interventions. Resistance may arise through extracellular modifications, including structural alterations or loss of cell-surface receptors, secretion of extracellular polymeric substances, and the release of outer membrane vesicles, as well as quorum-sensing–mediated regulatory shifts [153]. In addition, intracellular defense systems, such as restriction–modification, CRISPR-Cas immunity, abortive infection pathways, superinfection exclusion, and recurrent infection cycles, further restrict phage replication [154]. These mechanisms reflect an ongoing evolutionary arms race in which bacterial immune systems exert continuous selective pressure on therapeutic phages, leading in some cases to rapid emergence of phage-insensitive mutants and treatment failure, as noted in recent gastrointestinal and broader phage therapy analyses [155,156].

Although certain phages can adapt by modifying receptor-binding proteins to recognize new bacterial targets, resistance remains a major limitation for sustainable therapeutic use. Moreover, such resistance may involve fitness costs for bacteria, such as reduced growth or virulence, yet these disadvantages are not always sufficient to ensure clinical success. To address this, several mitigation strategies have been proposed, including the design of phage cocktails targeting multiple receptors [156,157], the ongoing isolation of novel phages capable of bypassing emergent resistance [158,159], and the genetic engineering of phages to overcome bacterial defense systems [160,161]. For example, Thanki Anisha et al. [162] demonstrated that a three-phage cocktail (CPLM2, CPLM15, and CPLS41) significantly reduced *C. perfringens* colonization in a larval infection model. Nonetheless, such approaches introduce manufacturing, regulatory, and logistical complexities, particularly in large-scale applications such as poultry production, underscoring the need for resistance-aware therapy design.

### 8.2. Phage Selection and Effective Delivery

Successful bacteriophage application depends on rigorous selection and reliable delivery to the site of infection. Therapeutic candidates must demonstrate the ability to infect and replicate within target bacteria through a strictly lytic life cycle, and phages encoding toxin genes, antibiotic resistance determinants, recombinases, or integrases are excluded to ensure safety. Expert recommendations emphasize assembling cocktails composed of genetically diverse phages with broad host ranges, high adsorption efficiency, and stability across variable physicochemical environments, as these characteristics enhance therapeutic robustness and reduce the likelihood of treatment failure [163]. Meeting such criteria during early selection is essential, particularly given ongoing challenges in identifying suitable phages for specific pathogenic strains, manufacturing constraints, and the risk of resistance emergence [164]. These issues are compounded by limited scientific evidence, the scarcity of randomized clinical trials, inadequate large-scale production infrastructure, and the absence of harmonized global regulatory and quality standards, which disproportionately affect low-income regions [44].

Beyond selection, ensuring that phages reach and remain active at the intended anatomical site remains a major practical barrier. For instance, surface application on meat, eggshells, or processed foods can reduce *Salmonella* contamination, but preventing intestinal colonization in poultry requires oral administration. Within the gastrointestinal tract, exposure to highly acidic conditions may inactivate phages lacking sufficient acid tolerance. Encapsulation approaches, such as microencapsulation, liposomal systems, and dry or liquid formulations, provide a feasible strategy to enhance phage survival, protect against gastric acidity, and improve stability and delivery efficiency during gastrointestinal transit [165]. Together, these considerations highlight that effective phage therapy relies not only on selecting appropriate viral candidates but also on overcoming regulatory, logistical, and biological challenges associated with their deployment.

### 8.3. Phage Behavior in Complex Microbiomes and In Vivo Systems

In real-world settings, microbial ecosystems such as the gastrointestinal tract or the poultry gut are highly diverse and dynamic, and the interactions between therapeutic phages and their bacterial targets become more complex than in simplistic in vitro mono-culture systems. Endogenous phages and the resident bacterial community exert ecological pressures that can affect the successful adsorption, replication, and lysis of exogenously administered phages. In the human gut, studies have shown that temperate phages contribute to community structure, lysogeny, and lateral gene transfer, thereby modulating bacterial phenotypes and influencing susceptibility to external phage attack [166]. Mechanistically, phage infection in a microbiome must contend with competition for adsorption sites, bacterial physiological states (dormant, biofilm-associated, low-density) that reduce phage burst size, and spatial structuring (e.g., mucosal layers, biofilms) that limit phage diffusion. Phage–phage interactions, including superinfection exclusion and competition for the same bacterial receptor, may further reduce the effective host range of phage cocktails, an issue often overlooked in simplified formulations [167]. Consequently, the narrow host range of an individual phage may be even more constrained in situ, and cocktails may fail to broaden coverage additively due to antagonistic interactions or ecological barriers. Addressing these dynamics requires more elaborate phage selection, cocktail design, and monitoring of replication kinetics within complex microbiomes.

A key technical bottleneck for phage therapy is in vivo stability, biodistribution, and clearance kinetics, which differ substantially from conventional antibiotics. After systemic administration, phages encounter immune neutralization, sequestration by the reticulo-endothelial system, complement-mediated inactivation, antibody responses, and rapid clearance from circulation. For example, in a sheep model, intravenous phages were eliminated from circulation within 240 min, and neutralizing antibodies reached approximately 99.9% by day 48, severely restricting therapeutic persistence [168]. Reviews of phage pharmacokinetics emphasize that standard absorption, distribution, metabolism, and elimination parameters must be considered, yet they often remain uncharacterized. For example, phage elimination may depend on phage size, surface proteins, opsonization, and splenic or hepatic uptake, factors more comparable to nanoparticle clearance than small-molecule antibiotics [169]. The interplay between passive dosing and in situ replication further complicates pharmacodynamic modeling; successful active therapy requires bacterial densities above a defined proliferation threshold [170]. Similar challenges arise in poultry infections, where oral, intramuscular, or spray-based delivery must overcome acidic gastric environments, mucosal barriers, and immune surveillance. Encapsulation strategies, including microencapsulation and liposomal formulations, can mitigate gastric or enzymatic degradation, but in vivo bioavailability, replication kinetics, and tissue penetration remain highly variable, and data in relevant animal models are still limited.

### 8.4. Environmental Release, Biosafety, and Phage Pharmacokinetics and Pharmacodynamics (PK/PD) Challenges

Environmental release of bacteriophages in livestock, agriculture, and food processing introduces ecosystem-level biosafety concerns alongside substantial challenges in phage pharmacokinetics and dosing. When phages are applied to poultry, eggs, water, feed, or farm environments, large quantities of viral particles enter open ecological systems. This release increases the likelihood of interactions with non-target bacteria, horizontal gene transfer, shifts in environmental microbiomes, and new selection pressures. Mechanistically, the use of temperate or insufficiently characterized phages raises the risk of transduction of undesirable genes such as toxins or antibiotic resistance determinants. Environmental exposure also promotes the emergence of phage-resistant bacteria, which may later colonize animals or humans and compromise therapeutic effectiveness. Regulatory analyses, including those from the EU’s Joint Research Centre, highlight the need to investigate the ecological impact of extensive phage use, particularly regarding resistance emergence and persistence [171]. Experimental and modeling research further indicates that structured environments such as soil, litter, fecal matter, and biofilms restrict phage diffusion, potentially fostering resistance development in non-target microbial populations [172]. In poultry biocontrol settings, these factors require systematic evaluation of phage selection (strictly lytic, genetically defined), risks of horizontal gene transfer, off-target effects on commensal and environmental communities, and the possibility of creating reservoirs of phage-resistant organisms.

In parallel, substantial variability in phage pharmacokinetics and pharmacodynamics complicates dosing standardization and prediction of therapeutic outcomes. Unlike antibiotics, which follow established PK/PD indices, phages exhibit predator–prey dynamics and replicate only when sufficient host bacteria are present. Replication kinetics are shaped by bacterial density, physiological state, immune-mediated clearance, and adsorption efficiency. Studies such as Kang et al. [173] emphasize that phage PK/PD is strongly dictated by the infection environment, while Bosco et al. [174] demonstrate wide host-to-host differences in neutralizing antibodies, clearance rates, and tissue distribution. For poultry applications, defining effective doses, timing, routes of administration, and formulations remains difficult. Delivery through feed or water often results in substantial phage loss due to pH, enzymatic degradation, or adsorption to non-target surfaces before reaching the gut. Once in the gastrointestinal tract, phages must encounter adequate target bacteria to initiate replication; if bacterial densities are low, the expected self-amplification does not occur, necessitating extremely high starting doses. These requirements introduce practical constraints related to cost, manufacturing feasibility, and potential immunogenicity. Overall, environmental biosafety risks and PK/PD variability represent critical bottlenecks that demand rigorous investigation and risk assessment before phage-based interventions can be reliably deployed in agricultural systems.

### 8.5. Regulatory Concerns and Limitations

Phages have been classified as pharmaceuticals and medicinal products in both the United States (US) and the European Union (EU) since 2011. In the US, the Food and Drug Administration (FDA) regulates bacteriophages within the food safety sector and has designated several phage preparations as Generally Recognized as Safe (GRAS), including those used to control *L. monocytogenes* in ready-to-eat meat products. In the EU, phages intended for use as food or feed additives must be evaluated by the European Food Safety Authority (EFSA), whereas therapeutic applications fall under the jurisdiction of the European Medicines Agency (EMA) [175]. Although no phage-based veterinary drugs have yet received full authorization in the EU, certain preparations may be used under experimental or compassionate-use frameworks. Several factors contribute to the regulatory challenges that hinder the global development and adoption of phage-based alternatives or adjuncts to conventional antibiotics. Phage therapy remains insufficiently understood, largely due to the limited number of clinical trials that meet national and international ethical standards [176,177]. Commercialization requires adherence to specific regulatory pathways, which vary depending on whether phages are intended for use as feed additives, disinfectants, or therapeutic agents. Developers must provide robust evidence of product safety and efficacy before marketing approval can be granted. There is an urgent need to address foundational safety considerations to build confidence in the use of bacteriophages as antimicrobial agents [178]. Table 5 summarizes commercially available and clinical-stage bacteriophage products, highlighting their primary applications and current regulatory or availability status. However, regulatory frameworks for phage products vary widely across countries, and no global consensus has been reached regarding their use in the livestock industry. Consequently, substantial additional research and regulatory harmonization efforts are still required.

## 9. Future Prospects of Bacteriophage Therapy

Bacteriophage therapy holds substantial promises for addressing antibiotic resistance in crops, animals, and humans. Growing interest in the use of phages as food antimicrobials has accelerated research into the efficacy of single-phage preparations and phage cocktails targeting specific bacterial pathogens, with minimal impact on human health. The unique antibacterial properties of phages and their advantages over conventional antibiotics continue to drive the development of innovative phage-based products. Emerging research areas, including engineered phages for drug delivery, combined phage–enzyme applications, genetic modifications to enhance therapeutic efficacy, and integrated phage–antibiotic strategies, are expected to significantly influence both veterinary and medical treatment modalities [55].

Antibiotic resistance is particularly prevalent in less developed countries due to insufficient healthcare infrastructure, unregulated agricultural practices, poor hygiene and sanitation, and widespread misuse of antibiotics. Lytic bacteriophages selectively eliminate pathogenic bacteria while sparing the gut microbiota and the host. Their rapid multiplication, cost-effectiveness, and environmental sustainability position phage-based solutions as viable tools for combating multidrug-resistant bacteria in these regions. Phage-mediated control of infectious diseases may also yield economic benefits in developing nations, where bacterial infections in livestock cause substantial financial losses. Because the physiological and genetic characteristics of pathogens vary by region, phage cocktails tailored to local bacterial strains can effectively reduce disease burden and associated economic pressures. Furthermore, the growth of biotechnology companies specializing in phage technology in these countries has the potential to generate new bioproducts, create employment opportunities, and strengthen global competitiveness. Such innovation could contribute meaningfully to addressing infectious disease challenges in livestock worldwide [193].

## 10. Conclusions

Efforts to enhance sustainability and food safety in poultry production increasingly rely on alternatives to conventional antibiotics, and bacteriophage therapy has re-emerged as one such candidate. Evidence from controlled trials and field investigations indicates that phage preparations can reduce the burden of enteric pathogens such as Campylobacter, *Salmonella*, *Escherichia coli*, and *Clostridium perfringens*, with measurable impacts on foodborne transmission risks and flock health. These findings demonstrate that phages can function as useful components of integrated pathogen-control strategies, particularly when applied under conditions that support adequate host contact, stability, and replication.

Despite these advantages, the therapeutic and prophylactic performance of phages remains constrained by several biological and operational limitations. Host specificity, variability in in vivo pharmacokinetics, interactions within complex microbiomes, and the capacity of bacteria to rapidly evolve phage resistance all influence their reliability. Environmental and regulatory considerations further shape the feasibility of routine implementation in livestock systems. Comparative studies show that phages are most effective when used in combination with complementary interventions, including biosecurity practices, vaccination, competitive exclusion products, and improved management of antimicrobial use. Their role is therefore best understood as adjunctive rather than universally substitutive.

Advances in genomics, structural biology, and synthetic engineering are progressively improving the safety, precision, and adaptability of phage preparations. These technologies enable more rigorous characterization of phage genomes, more predictable host-range profiles, and the development of engineered phages capable of circumventing dominant bacterial defense systems. However, translating these innovations into reliable commercial products requires coordinated progress in production standards, regulatory evaluation, and large-scale efficacy testing.

Taken together, current evidence supports the potential of bacteriophages as targeted tools for reducing specific pathogens in poultry production, while also underscoring the need for careful application and realistic expectations. Rather than positioning phages as a standalone replacement for antibiotics, the available data indicate that they are most effective as part of an integrated, evidence-based approach to antimicrobial stewardship and food safety. Continued research, regulatory harmonization, and systematic field validation will determine the extent to which phage therapy can contribute sustainably and safely to modern poultry health management.

## Figures and Tables

**Table 5:** Regulatory status of commercially marketed bacteriophage-based products relevant to enteric bacterial infections in poultry. Adapted and modified from [42,55,179].

Phage Product	Company	Target Organism (s)	Regulatory	References
ListShield^™^	Intralytix, Inc. (Baltimore, MD, USA)	*L. monocytogenes*	FDA, 21 CFR 172.785; FDA, GRN 528; EPA Reg. No. 74234-1; Israel Ministry of Health; Health Canada	[180,181]
ShigaShield^™^ (ShigActive^™^)	Intralytix, Inc. (Baltimore, MD, USA)	*Shigella* spp.	FDA, GRN 672	[182]
EcoShield™	Intralytix, Inc. (Baltimore, MD, USA)	*E. coli* O157:H7	FDA, FCN 1018; Israel Ministry of Health; Health Canada	[183,184,185,186,187]
Ecolicide^®^ (EcolicidePX^™^)	Intralytix, Inc. (Baltimore, MD, USA)	*E. coli* O157:H7	USDA, FSIS Directive 7120.1	[55]
SalmoFresh™	Intralytix, Inc. (Baltimore, MD, USA)	*Salmonella* spp.	FDA, GRN 435; USDA, FSIS Directive 7120.1; Israel Ministry of Health; Health Canada	[125]
Secure Shield E1	FINK TEC GmbH (Hamm, Germany)	*E. coli*	FDA, GRN 724 pending as of 19 March 2018	[55]
PhageGuard S^™^	Micreos Food Safety (Wageningen, The Netherlands)	*Salmonella* spp.	FDA, GRN 198/218; FSANZ; EFSA; Swiss BAG; Israel Ministry of Health; Health Canada	[126,188]
PhageGuard Listex^™^	Micreos Food Safety (Wageningen, The Netherlands)	*L. monocytogenes*	FDA, GRN 198/218; FSANZ; EFSA; Swiss BAG; Israel Ministry of Health; Health Canada	[189,190]
Finalyse^®^	Passport Food Safety Solutions (West Des Moines, IA, USA)	*E. coli* O157:H7	USDA, FSIS Directive 7120.1	[191]
AgriPhage^™^	Phagelux (Shanghai, China)	*Xanthomonas campestris* pv. vesicatoria, *Pseudomonas syringae* pv. tomato	EPA Reg. No. 67986-1	Intralytix Corp. Website
SalmoPro^®^	Phagelux (Shanghai, China)	*Salmonella* spp.	FDA, GRN 603 FDA, GRN 752 pending as of 19 March 2018	[152]
SalmoPro^®^	(Montreal, QC, Canada)	*Salmonella enterica*	FDA-approved, granted GRAS status	[123]
Bafasal^®^	Proteon Pharmaceuticals (Łódź, Poland)	*Salmonella*	Regulatory-approved feed additive	[122]
Biotector^®^ S	CJ CheilJedang Research Institute of Biotechnology (Seoul, South Korea)	*Salmonella* Gallinarum, *Salmonella* Pullorum	can be applied to animal feed to control *Salmonella* in poultry	[123]
Salmonelex™ (PhageGuard)	Micreos Food Safety BV (The Netherlands)	*Salmonella*	FDA-approved, granted GRAS status	Phage Guard Corp.
BacWash^TM^	OmniLytics Inc. (Sandy, UT, USA)	*Salmonella*	for disinfection of surfaces	[124]
SalmoFREE^®^	Sciphage (Bogotá, Colombia)	*Salmonella*	for therapy and control of *Salmonella* in poultry farms	[86]
Listex™ P100 (PhageGuard)	Micreos Food Safety BV. (Wageningen The Netherlands)	*L. monocytogenes*	FDA-approved, granted GRAS status	[175]
SalmoLyse^®^	USA based	*Salmonella* spp.	FDA	[192]
PLSV-1™	USA based	*Salmonella* spp.	FDA	[55]
INT-401™	Intralytix Corp. (Baltimore, MD, USA)	*C. perfringens*	FDA, FSIS	Intralytix Corp. Website

FDA, U.S. Food and Drug Administration; GRAS, generally recognized as safe; BAG, Bundesamt für Gesundheit; CFR, Code of Federal Regulations; FSIS, Food Safety and Inspection Service; GRN, GRAS Notice.

## Data Availability

No new data were created or analyzed in this study. Data sharing is not applicable to this article.
